# Dementia assessment and diagnostic practices of healthcare workers in rural southwestern Uganda: a cross-sectional qualitative study

**DOI:** 10.1186/s12913-019-4850-2

**Published:** 2019-12-27

**Authors:** Ronald Kamoga, Godfrey Z. Rukundo, Edith K. Wakida, Gladys Nakidde, Celestino Obua, Stephanie S. Buss

**Affiliations:** 1 Mbarara Alzheimer’s and Related Dementias Research Initiative (MADRI) fellow, Department of Anatomy, Faculty of Medicine Mbarara University of Science and Technology, P.O. Box 1410, Mbarara, Uganda; 2 Department of Psychiatry, Faculty of Medicine, Mbarara University of Science and Technology, P. O. Box 1410, Mbarara, Uganda; 3 Office of Research Administration, Faculty of Medicine, Mbarara University of Science and Technology, P. O. Box 1410, Mbarara, Uganda; 4 Department of Nursing, Faculty of Applied Science, Bishop Stuart University, P. O. Box 09, Mbarara, Uganda; 5 Department of Pharmacology, Mbarara University of Science and Technology, P. O. Box 1410, Mbarara, Uganda; 6 Beth Israel Deaconess Medical Center, Harvard Medical School, Boston, MA USA

**Keywords:** Dementia, Uganda, Assessment, Diagnosis, Healthcare workers, Rural

## Abstract

**Background:**

An estimated 50 million people worldwide have Alzheimer’s disease and related dementias (ADRD), and this number is projected to increase with the growth of the aging population, with the largest growth occurring in low and middle-income countries. Diagnostic coverage for dementia is estimated to be only 5–10% in low- and middle-income countries. Timely diagnosis of ADRD could prompt early access to information, medical treatments, and support for caregivers. The aim of this study was to assess how healthcare workers in rural southwestern Uganda assess for and diagnose ADRD**.**

**Methods:**

We used in-depth interviews to investigate the medical knowledge and clinical practices surrounding ADRD diagnoses among 42 healthcare workers employed at mid-tier health facilities in southwestern Uganda. Qualitative content analysis was used to identify distinct categories and themes.

**Results:**

Our findings show that healthcare workers without specific mental health training assessed and diagnosed dementia based on history and physical examination alone. On the other hand, healthcare workers with some specialized training in mental health were more likely to use neuropsychological tests, blood tests, urine tests, and brain imaging in the diagnosis of dementia. Collateral history from caregivers was noted to be very important in proper assessment and diagnosis of dementia among all categories of healthcare workers. The majority of healthcare workers regarded memory loss as part of the normal aging process and reported that it does not need any specific treatment. Other healthcare workers could recognize signs and symptoms of dementia, but focused on managing other medical problems at the expense of assessing cognitive decline and mental health. Diagnostic practices did not differ based on age, years of experience, or gender of the healthcare workers.

**Conclusion:**

These results indicate that specialized training in mental health among healthcare workers is crucial for the assessment and diagnosis of ADRD in rural southwestern Uganda.

## Background

Alzheimer’s disease and related dementias (ADRD) affect approximately 50 million people worldwide, and this number is currently increasing due to the growth of the aging population, with the global prevalence expected to reach 152 million by 2050 [1]. Most of this increase is projected to occur in low- and middle-income countries (LMIC) [1–3]. Over 58% of people with ADRD currently live in LMIC including Sub-Saharan Africa, where more than 1.69 million people are estimated to be living with dementia [4]. ADRD is the most important independent cause of disability among the older people in LMIC [5].

Despite availability of numerous dementia diagnostic tools, 62% cases of ADRD worldwide are undiagnosed, while 91% are diagnosed very late [3, 6–8]. Missed or delayed diagnosis magnifies the socioeconomic burden on families and healthcare systems through heavy expenditures on unnecessary investigations, treatments driven by vague symptoms, and lack of counseling of family and caregivers [7, 9–12]. Early diagnosis could prompt early evaluation for reversible dementia syndromes as well as timely access to information, medical treatment, advice and support for caregivers, and targeted community interventions from the time of diagnosis to end of life [10, 13, 14].

Consensus guidelines on the assessment for ADRD includes medical history, physical examination, laboratory tests, cognitive assessment, and brain imaging [15]. Prior case reports have described the assessment and diagnosis of ADRD in sub-Saharan Africa and Uganda [16–18], but little is known about broader trends in dementia assessment and treatment in rural primary care settings in Uganda. The aim of the study was to assess how healthcare workers in health facilities in rural southwestern Uganda assess for and diagnose ADRD.

## Methods

### Study design

This was a cross-sectional qualitative study that used individual in-depth interviews to investigate how healthcare workers in health facilities in rural southwestern Uganda assess for and diagnose ADRD.

### Study setting

The study was conducted in the districts of Kabale, Rukungiri, and Mbarara, which are rural regions southwest of the capital city Kampala, Uganda. The districts were purposively selected based on the number of health facilities per district to ensure that an adequate number of participants could be interviewed. Kabale district had the highest number of healthcare facilities (*n* = 139), followed by Rukungiri (*n* = 88), and Mbarara (*n* = 58) [19].

In Uganda, dementia is regarded as a mental health condition. Provision of mental health services begin at health center III (HC III) which is sub-county level, with subsequent referrals to HC IV at the county level, district hospitals, regional referral hospitals, and finally to Butabika Hospital, the national referral hospital [20–22]. At HC IV level and above, medical facilities are expected to have general doctors (medical officers), clinical officers (diploma level medical assistants), nurses, midwives, and specialized mental health workers (psychiatric nurses, psychiatric clinical officers, and psychiatrists). The HC IIIs do not have general doctors or mental health specialists but have all the other cadres of healthcare providers. In our study, assessments were performed at mid-tier medical facilities: HCs III, HCs IV, general hospitals, and regional referral hospitals.

### Study population and sampling

The study participants included nurses, clinical officers, medical doctors and mental health specialists directly involved with assessment of patients in the participating health centers regardless of gender, age, and seniority. From each district, we purposively selected participants from one hospital, 2 HC IVs, and 2 HC IIIs based on their active involvement in patient assessment. Representation from the different categories of healthcare workers was a key consideration in the selection of participants in order to understand how different categories of healthcare workers assess and diagnose dementia.

We recruited participants until saturation was reached. Saturation was considered by consensus within the team as the point when no new information or emerging themes were obtained from each category of study participants with subsequent interviews. Saturation was reached at seven for medical officers, at 12 for clinical officers, at 15 for nurses, and at eight for specialized mental health workers. For the purposes of analysis in this study, a specialized mental health worker was defined as anyone with special training in mental health including a psychiatric nurse, psychiatric clinical officers, psychiatrist, or any other medical professional with extra training in mental health conditions.

### Data collection tools

Data collection was done through in-depth face to face interviews using open-ended questions in line with the study objectives. An interview guide (Table 1) was developed by RK together with GZR and SB to assess basic care knowledge and practice expected from a general healthcare worker in a rural setting. The questions in the interview guide were based on literature and the republic of Uganda Ministry of Health Uganda Clinical Guidelines manual [21].

**Table 1:** The Interview guide used to get in-depth insight in how health workers assessed and diagnosed dementia is shown. Semi-structured open-ended questions are designated in brackets

Biographic data: Job title, level of training, Gender, Age, Number of years in post	
Approximately how many elderly people, around 60 years and older do you see at this health center per day?	
What have you observed about the behavior of your elderly patients? (Probe for difficulty remembering history, “odd” behavior, poor hygiene, inappropriately dressed for the weather.)	
How comfortable are you in assessing elderly patients suspected to have dementia? (Probe for how they asses memory loss, difficulty performing familiar tasks, problems with language, disorientation to time and place, poor or decreased judgment, problems with abstract thinking, misplacing things, changes in mood or behavior, changes in personality, loss of initiative.)	
Please tell me about the kind of tools used to assess for dementia here at your health facility? (Probe for whether they perform a cognitive examination or neurological examination, and whether they use the MOCA, MMSE, or other standardized assessment tool.)	
Tell me about any training you have received about assessing for dementia? (Probe for what sorts of training would they want to get, if they wanted more training for dementia.)	
Do you commonly recommend any diagnostic tests for someone suspected of having dementia? (Probe for whether they recommend lumbar puncture; blood work such as HIV, RPR, B12, CBC, LFTs; depression screening; neuroimaging.)	
If a patient is suspected of having dementia, do you commonly tell them their diagnosis? Do you tell their family or caregiver the diagnosis? (Probe for whether they integrate family members; prepare anything for disclores meeting; consider the patient’s perspective or prepare for the patient’s or family’s reaction to the diagnosis)	

*MOCA* Montreal-Cognitive Assessment, *MMSE* Mini Mental State Examination, *HIV* Human Immunodeficiency Virus, *RPR* Rapid Plasma Reagin, *B12* Vitamin B12, *CBC* Cell Blood Count, *LFTs* Liver Function Tests

### Data collection procedure

In-depth interviews were conducted by the lead author and three trained research assistants between January and February 2019. Each interview was conducted until there was no new information generated. Interviews lasted approximately 40 min. All interviews were conducted in English, the official national language. They were audio recorded and backed by field notes with participant’s consent. Two research teams were used to collect data concurrently. Each team of two researchers interviewed a maximum of three participants per day. One member conducted the interview while the other was recording the interview with an audio recorder and taking notes. The team members alternated roles with subsequent interviews.

### Quality control

The lead author RK and research assistants were trained on study objectives, informed consent, ADRD epidemiology, and data collection procedures. Interview guides were pilot tested at a separate health facility for usability and clarity. There were feedback meetings at the end of each day of data collection to check for completeness of the data. GZR provided oversight during the feedback process and attended some of the interviews to ensure that interviews were conducted properly according to the study objectives.

### Data management and analysis

Field data collected from interviews was immediately transcribed verbatim and cleaned by GN, and checked by the lead author RK against the audio recordings for correctness of information before proceeding to the next set of interviews. Processed data were then entered into Atlas.ti version 7 (qualitative analysis software for Windows) for coding and analysis. Codes were developed based on the broad themes identified from the data by RK in consultation with GN, SB, GZR, and CO. The individual team members each independently formulated codes based on emerging themes from the data before they then met as a team to discuss the codes and emerging themes from the data. A codebook was formed, presented and discussed by the team, and finalized for use in analysis. RK then read through all the transcripts several times and applied the codes to each section of data collected. The codes were used to retrieve segments of the data using the Atlas.ti software. Memos were written describing the patterns and variations in the different segments of retrieved data. Data matrices were drawn out of the retrieved data sets which were then organized into broad themes which mirrored the study objectives. Emerging patterns in the data were described with rich verbatim quotes from participants to illustrate the key issues in the themes. The computer-assisted analysis of data helped to ensure standardized and comparable analysis and interpretation of qualitative data across different levels of training and gender of participants.

Confidentiality was ensured by allocating non-identifiable field codes to each participant in addition to conducting the interviews in private space. We respected individual autonomy to participate in the study and did not include those who declined to participate. All who consented to participate were informed about their freedom to withdraw from the study at any time without any penalties.

## Results

The three broad themes emerged from the findings including (a) clinical assessment skills, (b) use of cognitive tests, and (c) use of diagnostic studies. Representative quotes have been included, followed in brackets by gender of participant, level of mental health training and district of the participants.

A total of 42 participants were interviewed; demographic characteristics are summarized in Table 2. Specialized mental health workers included two psychiatric clinical officers, three psychiatric nurses, one general clinical officer, one medical officer, and one general nurse.

**Table 2:** The characteristics of participating health workers. A subset of participants in each training category had received specialized training in mental health

	Medical Officer	Clinical Officer	Nurse
Level of training	Bachelor of Medicine and Surgery	Diploma in general clinical medicine, Diploma in clinical psychiatry, or Short course in mental health	Diploma in nursing or Diploma in psychiatric nursing
Age range (years)	28–44	26–50	25–58
Years of experience	1–6	1–18	2–13
Gender			
Male	5	11	2
Female	3	4	17
Special training in mental health	1	3	4

### Clinical assessment skills

All participants regardless of level of training, gender, and years of experience highlighted the importance of collateral history by caregivers in the assessment of older patients presenting with signs and symptoms of dementia.

***“****Majorly we always ask their (dementia patients’) attendants or care takers for information that can lead to meaningful diagnosis” (Male, Clinical officer, Mbarara District)*

Specialized mental health workers reported that their prior training in mental health was crucial for the proper assessment and diagnosis of dementia.

*“We used to be so green about them (signs and symptoms of dementia), but last year we had a training about assessing mental health patients and now we are somehow catching up. Actually, that time we never used to do (even) counseling. We could always say this category of people (older patients with dementia signs) is always like that. But now we got some light, now we even see it (dementia) as a condition.” (Female, General Nurse, Mbarara District)*

*“Me myself am comfortable (assessing older patients with signs of dementia) maybe because of my background. I never started as a medical officer. I was an educational therapist so I had worked in the psychiatric setting. But for others who don’t have training in that area (mental health), I think it is more of a challenge.” (Male Medical officer with prior training in mental health, Kabale district).*

On the other hand, participants without specialized mental health training reported discomfort and uncertainty of their knowledge and skills in assessing a patient with signs of dementia.

*“....to be honest, I don’t like such patients (patients with dementia). I have deficient knowledge of assessment and skills (for people with dementia) but I go on to assess (them) because it a job to do...” (Female Clinical officer, no specialized training in mental health, Rukungiri district)*

Some participants without specialized mental health training perceived signs and symptoms of dementia as signs of a normal aging process, and stated that nothing could be done about it.

*“ … to be honest I usually don’t consider memory loss, in especially that age group you have talked about (the older patients), as something that is worrying. So, I tend to actually give more attention to the physical symptoms and signs that I can pick out because that one (memory loss), I know it’s part of the aging process.” (Female Medical officer, Rukungiri district)*

*“ … … No really because sometimes I take it for granted that it’s because of the age and I assume that I can’t help much because old age cannot be treated” (Male Clinical officer, Mbarara district)*

Many healthcare workers without mental health training expressed the notion that they observe many older people with signs and symptoms of dementia but they feel unable to do anything. These participants reported that they either ignore the signs or look for and treat other comorbidities in these patients. Some expressed the impression that they were completely ignorant while others admitted to having very deficient knowledge and skills to assess and diagnose dementia. The later explained that the training in medical schools did not emphasize importance of diagnosing or treating dementia. Some speculated this was because of a perception of the rarity of dementia in clinical practice, while others thought that their lecturers may have also lacked confidence in their own knowledge related to dementia. Due to a lack of knowledge about dementia, healthcare workers reported that they chose to look for and treat other comorbidities so that they could at least offer some sort of diagnosis and treatment to patients who came seeking medical care in their clinics. No participant mentioned the fear of labeling a patient with dementia (stigma) due to its negative perceptions in society as a cause for looking for and treating other comorbidities.

*“I just overlook them (signs of dementia) because even I don’t know what to do with it (dementia)”* (*Female Clinical officer, no specialized training in mental health, Kabale district)*

*“ … … but truth is, we get many clients (older patients with signs of dementia) were if someone appreciated the mental state exam, I think he would actually help a number of clients. Because we get them but we always somehow look for other things to manage and not do a lot to address mental state issues even though we actually see that we have people in need.” (Male Medical officer, no specialized training in mental health, Mbarara district)*

Differences in gender and years of experience did not have an effect on how participants assessed and diagnosed dementia.

### Use of cognitive tests

Specialized mental health workers were more likely to use standardized assessment tools such as Mini Mental Status Exam (MMSE). Many healthcare workers without mental health training reported to have no special tools for assessing dementia in suspected patients apart from the general clinical assessment methods of history and physical examination. These participants reported that they were not familiar with the standard procedures used in assessing dementia and they had not participated in any refresher trainings in mental health. These findings held across participants regardless of the level of training, gender, and years of experience.

*“I will not lie; we don’t (have any tool). At least if we do, I have never interfaced with it in this hospital. So basically, we do basic general assessments. We do not really have a protocol well set out or established here that confirms or safe guidelines that we use to assess and determine this condition (dementia)” (Medical officer, no specialized training in mental health, Male, Mbarara district)*

Other healthcare workers were aware that the MMSE tool existed but they felt inadequately skilled to apply it in assessing older adults suspected to have dementia.

*“You always don’t tend to think that we might benefit a lot from mental status exam or even then, the expertise to do a mental state exam is actually lacking very significantly.” (Male Medical officer, no specialized training in mental health, Mbarara district)*

Apart from the MMSE, there was no other dementia assessment tool mentioned or known to any of the healthcare workers who participated in the study.

### Use of diagnostic tests

The use of diagnostic tests also varied according to level of training of healthcare workers. All of the specialized mental health workers and some of the other healthcare workers were able to mention relevant investigations to rule out other possible causes of dementia. The most common tests mentioned by the participants included tests for malaria, syphilis, human immunodeficiency virus, serum electrolytes, urinalysis, complete blood count, liver function tests, and renal function tests, vitamin B12, blood sugar, and neuroimaging techniques (magnetic resonance imaging and computerized tomography scan). Neuroimaging techniques were mentioned by medical officers and clinical officers, but were not mentioned by nurses. A number of healthcare workers explicitly stressed the idea that it is not necessary to make any investigations for anyone with signs and symptoms of dementia.

*“If there is no other condition and I really feel its dementia, maybe it is just a knowledge gap but I don’t know any test. In me I don’t think any test will confirm dementia.” (Male Clinical officer, no specialized training in mental health, Mbarara).*

## Discussion

This study investigated how healthcare workers assess for and diagnose ADRD in mid-tier healthcare facilities in southwestern Uganda. Medical history, collateral history, physical examination, and use of Mini Mental Examination (MMSE) screening tool were identified by participants as the basis for diagnosing dementia. There was a notable difference in the assessment and diagnosis of dementia between specialized mental health workers and the other healthcare workers. Participants with specialized training in mental health were more likely to use standardized assessment tools or neuropsychological tests such as MMSE. Some participants believed that memory loss is part of a normal aging process that does not need any treatment. Other healthcare workers could recognize signs and symptoms of ADRD but do not pursue further work up because they are not sure of further steps in diagnosis or management.

In our study, only healthcare workers with specialized training felt comfortable in making a clinial diagnosis of ADRD using clinical interview, cognitive exam, and ancillary tests. Consensus guidelines recommend that clinical diagnosis of dementia follow established criteria and require a clinical history from the patient and informants, standardized neuropsychological test such as the MMSE, and assessment for reversible causes of dementia and comorbid conditions [3, 23]. Our findings therefore suggest that a significant population of ADRD patients in southwestern Uganda are likely to go undiagnosed due to the fact that there is a small percentage of a healthcare workers with mental health training who have the knowledge and skills to use neuropsychological tests to assess for dementia. This is in line with findings from other LMIC, with estimates showing that up to 62–90% of people with dementia in developing countries are likely to be undiagnosed [7, 24]. Further epidemiological studies measuring the prevalence and diagnostic coverage of dementia in Uganda are needed.

Our results show that most general trained healthcare workers held the view that memory loss is a part of a normal aging process with no effective interventions available. This knowledge gap is likely to lead to delayed and missed diagnosis of dementia and a lost opportunity to offer help and support to patients and caregivers. Specialized mental health workers were unlikely to hold this view, further demonstrating and underlining the importance of mental health training in dementia assessment and diagnosis care. Our observation fits with data worldwide showing that both the general public and healthcare workers often viewed memory loss as a normal part of the aging process [25–28]. Therefore, policymakers and planners seeking to improve early detection and diagnosis of ADRD should offer dementia-focused mental health training for healthcare workers which includes improved professional awareness and understanding of dementia in addition to specific training in clinical diagnostic criteria, cognitive assessment, and use of ancillary tests.

### Study limitations

A limitation to this study is that we employed a qualitative study design using individual interviews with limited scope. Individual interviews are best suited to elicit experiences and beliefs, and qualitative content analysis allows investigators to gain in-depth understanding of the latent contents of conversations of informants, which differs from the goals of qualitative research studies [29–31]. Therefore, the data from the current study provides in-depth insights into the challenges of dementia assessment and diagnosis facing healthcare workers in rural southwestern Uganda. Additional studies in other communities worldwide are needed to determine the degree to which our findings generalize to other rural communities and their healthcare systems. Furthermore, our data highlights opportunities for future epidemiological studies to quantify dementia burden in rural Uganda, and lays the foundation for future interventional research projects seeking to improve ADRD assessment and diagnosis in Uganda and other LMIC.

## Conclusion

Training in mental health is very crucial for all healthcare workers in order to be able to recognize and respond appropriately to signs of dementia. Our findings point out key gaps in the knowledge and skills of healthcare workers in dementia assessment and diagnosis that should be targeted by future interventions aiming to improve assessment, diagnosis, and management of dementia by healthcare workers in southwestern Uganda and other resource-limited settings.

## Data Availability

The full dataset generated and analyzed during the current study are not publicly available in order to maintain the privacy of the individuals interviewed during this study. Deidentified data can be made available from the corresponding author on reasonable request.

## Abbreviations

ADRD: Alzheimer’s and Related Dementias
B12: Vitamin B 12
CBC: Cell Blood Count
HC: Health Center
HIV: Human Immunodeficiency Virus
LFTs: Liver Function Tests
LMIC: Low and Middle Income countries
MADRI: Mbarara Alzheimer’s Disease and Related Dementias Initiative
MMSE: Mini Mental State Examination
MOCA: Montreal-Cognitive Assessment
RPR: Rapid Plasma Reagin
